# Hypoperfusion of the infrapatellar fat pad and its relationship to MRI T2* relaxation time changes in a 5/6 nephrectomy model

**DOI:** 10.1038/s41598-021-89336-8

**Published:** 2021-05-11

**Authors:** Guo-Shu Huang, Yi-Jen Peng, Yu-Juei Hsu, Herng-Sheng Lee, Yue-Cune Chang, Shih-Wei Chiang, Yi-Chih Hsu, Ying-Chun Liu, Ming-Huang Lin, Chao-Ying Wang

**Affiliations:** 1 Department of Radiology, Tri-Service General Hospital, National Defense Medical Center, Taipei, Taiwan; 2 Department of Medical Research, Tri-Service General Hospital, National Defense Medical Center, Taipei, Taiwan; 3 Department of Pathology, Tri-Service General Hospital, National Defense Medical Center, Taipei, Taiwan; 4 Division of Nephrology, Department of Medicine, Tri-Service General Hospital, National Defense Medical Center, Taipei, Taiwan; 5 Department of Pathology and Laboratory Medicine, Kaohsiung Veterans General Hospital, Kaohsiung, Taiwan; 6 Department of Mathematics, Tamkang University, New Taipei, Taiwan; 7 Institute of Biomedical Sciences, Academia Sinica, Taipei, Taiwan; 8 Department and Graduate Institute of Biology and Anatomy, National Defense Medical Center, No.161, Sec. 6, Minquan E. Rd., Neihu Dist., Taipei, 11490 Taiwan

**Keywords:** Magnetic resonance imaging, Musculoskeletal system, Chronic kidney disease

## Abstract

The purpose of present study was to longitudinally investigate the alterations in infrapatellar fat pad (IPFP) vascularity in 5/6 nephrectomized rats by using dynamic contrast enhanced (DCE) MRI and IPFP degeneration by using MRI T2* relaxation time. Twelve male Sprague–Dawley rats were assigned to a control group and a 5/6 nephrectomy CKD group. The right knees of all rats were longitudinally scanned by 4.7 T MRI, and serial changes in the IPFP were assessed at 0, 8, 16, 30, and 44 weeks by DCE-MRI (parameters *A, k*_*el*_ and *k*_*ep*_) and MRI T2* mapping. After MRI measurements, knee specimens were obtained and evaluated histologically. The CKD group had IPFPs with lower blood volume *A* and lower permeability *k*_*ep*_ values from 16 weeks (*p* < 0.05), lower venous washout *k*_*el*_ value from 30 weeks (*p* < 0.001), and significantly higher T2* values reflecting adipocyte degeneration beginning at 16 weeks (*p* < 0.05). The histopathological results confirmed the MRI findings. Hypoperfusion and adipocytes degeneration related to CKD were demonstrated in a rodent 5/6 nephrectomy model. DCE parameters and MRI T2* can serve as imaging biomarkers of fat pad degeneration during CKD progression.

## Introduction

Chronic kidney disease (CKD) is a progressive condition leading to kidney function impairment and eventually renal failure^1,2^. The chronic kidney disease-mineral and bone disorder (CKD-MBD), a systemic disorder of mineral and bone metabolism due to CKD, manifests as calcium and phosphorus dysregulation, bone turnover disturbance, and vascular calcification^3^. A variety of musculoskeletal disorders were associated with it including renal osteodystrophy, osteoporosis, amyloid arthropathy and bone fractures^3^. Osteoarticular complications are common in patients with CKD and cause disability. Further implantation of arthroplasty due to OA such as hip and knee replacements may be needed at the end stage of CKD^4^. The prevalence of grade III or IV osteoarthritis (OA) in CKD patients aged 15–64 years (9.4%) was three times greater than in the normal population (3.0%). In hemodialysis patients, the incidence of OA is high (53.9%) and localized in all joints including the knee joint (24.7%)^5–7^. Moreover, the chronic musculoskeletal pain associated with CKD has mechanisms that are multi-factorial in nature^8^. The infrapatellar fat pad (IPFP) is regarded as having a pivotal role in the initiation and progression of knee OA, and is associated with pain severity^9^^.^ Whether the OA and CKD-related OA have a similar etiology or represent two different entities remains unclear. Since knowledge about IPFP’s function in CKD is limited and needs clarification, investigation into the IPFP might be helpful to understand the complex pathogenesis of the CKD-related OA and find the possible methods of treating the pain.

The IPFP is a high vascularity extrasynovial adipose tissue in the knee joint, in close contact with the synovium. It has a network of vessels with numerous anastomoses and is supplied by a network of genicular arteries surrounded by fibrous tissue^10^. Meanwhile, it is a source of cytokines such as interleukin (IL)-6 and tumor necrosis factor (TNF)-α^11,12^, and adipokines^9^. Repetitive microtrauma after mechanical impingement of the IPFP may lead to ischemia, induce the abnormal distribution of substance P, and result in tissue inflammation or hemorrhage^13^. In regard to the pathogenesis of CKD-related OA, IPFP injury may develop and subsequently cause structural alteration of adipose tissue, or metabolic responses to adipocytokines. Accordingly, an examination of the change in IPFP might shed light on the pathophysiologic progression of CKD-related OA.

Magnetic resonance imaging (MRI) provides detailed insight into arthritis pathology and is a unique tool for evaluating the structural changes of IPFP^14,15^. Dynamic contrast-enhanced MRI (DCE-MRI) can be applied to extract perfusion parameters from the signal intensity curves of tissues and detect the distribution of the contrast uptake through signal-intensity curve analysis^16^. Semi-quantitative perfusion parameters can be easily measured, but provide limited information regarding the physiological processes affected. The Brix pharmacokinetic model is a robust two-compartment model suitable for tissues with slow perfusion rate^17^. Three perfusion parameters can be extracted such as the amplitude, *A*, the elimination constant of the contrast medium from the plasma, *k*_*el*_, and the exchange rate constant from the extravascular extracellular space (EES) to plasma, *k*_*ep*_. In previous reports, DCE-MRI has been used to study synovial vascularity and inflammation in diseases such as rheumatoid arthritis (RA)^18^ and osteoarthritis (OA)^19^, but few reports have examined its use to study the pathogenesis of CKD-related OA^20^. To clarify the hemodynamic response to CKD in the highly vascularized infrapatellar fat pad, longitudinal DCE-MRI was used to explore the role of perfusion at different stages of CKD. MRI T2* mapping has proven clinical utility as a tool for evaluating changes in the extracellular matrix composition of articular tissues including changes in hydration status and collagen^21,22^. In regard to the IPFP, a limited number of studies have proposed its potential as a reliable biomarker. Therefore, DCE-MRI and MRT T2* were used in our study to understand the pathophysiological processes underlying perfusion and compositional changes in IPFP after CKD.

We assumed that impaired vascularization and nutrient supply to the IPFP in CKD might be an important factor leading to IPFP degeneration. An animal model is needed to investigate longitudinal changes during CKD progression, and findings using this model are expected to apply to human beings in the future. Herein, the purposes of the present study were to (1) longitudinally investigate the relationship of DCE-MRI parameters and MRI T2* values to IPFP change in a rodent 5/6 nephrectomy model of CKD; (2) to assess the feasibility of using fat pad DCE-MRI parameters and MRI T2* values as imaging biomarkers of CKD-related OA progression; and (3) to analyse histopathological changes in IPFP. To the best of our knowledge this is the first study to assess infrapatellar fat pad changes as CKD progresses using longitudinal measurement of DCE-MRI parameters and MRI T2* values.

## Results

No adverse events were found. As shown in Table 1, the success of CKD induction 8 weeks after 5/6 nephrectomy was biochemically confirmed. For all rats, the inter-observer correlation coefficient was high (DCE: ICC = 0.967, MRI T2*: ICC = 0.909). The intra-observer correlation coefficient of the DCE and MRI T2* values was 0.995 and 0.908 respectively, indicating good reproducibility.

**Table 1:** Urine and serum biochemical data in control and CKD rats.

Urine or serum test	Control group	CKD group
**Urine**		
Color of sample	Colorless	Yellow
Protein (Pro)	48.33 ± 20.41	475.00 ± 150*
Urobilinogen (URO)	2.75 ± 0.95	3.00 ± 1.00
**Serum**		
Creatinine (Cr)	0.25 ± 0.06	0.80 ± 0.10*
Blood urea nitrogen (BUN)	13.95 ± 1.77	29.88 ± 5.05*
Calcium (Ca)	7.32 ± 1.30	8.80 ± 0.69
Phosphorus (P)	4.46 ± 0.35	7.83 ± 1.77*

All units: mg/dl.

*p value < 0.05.

### DCE-MRI analysis

**Figure 1:**
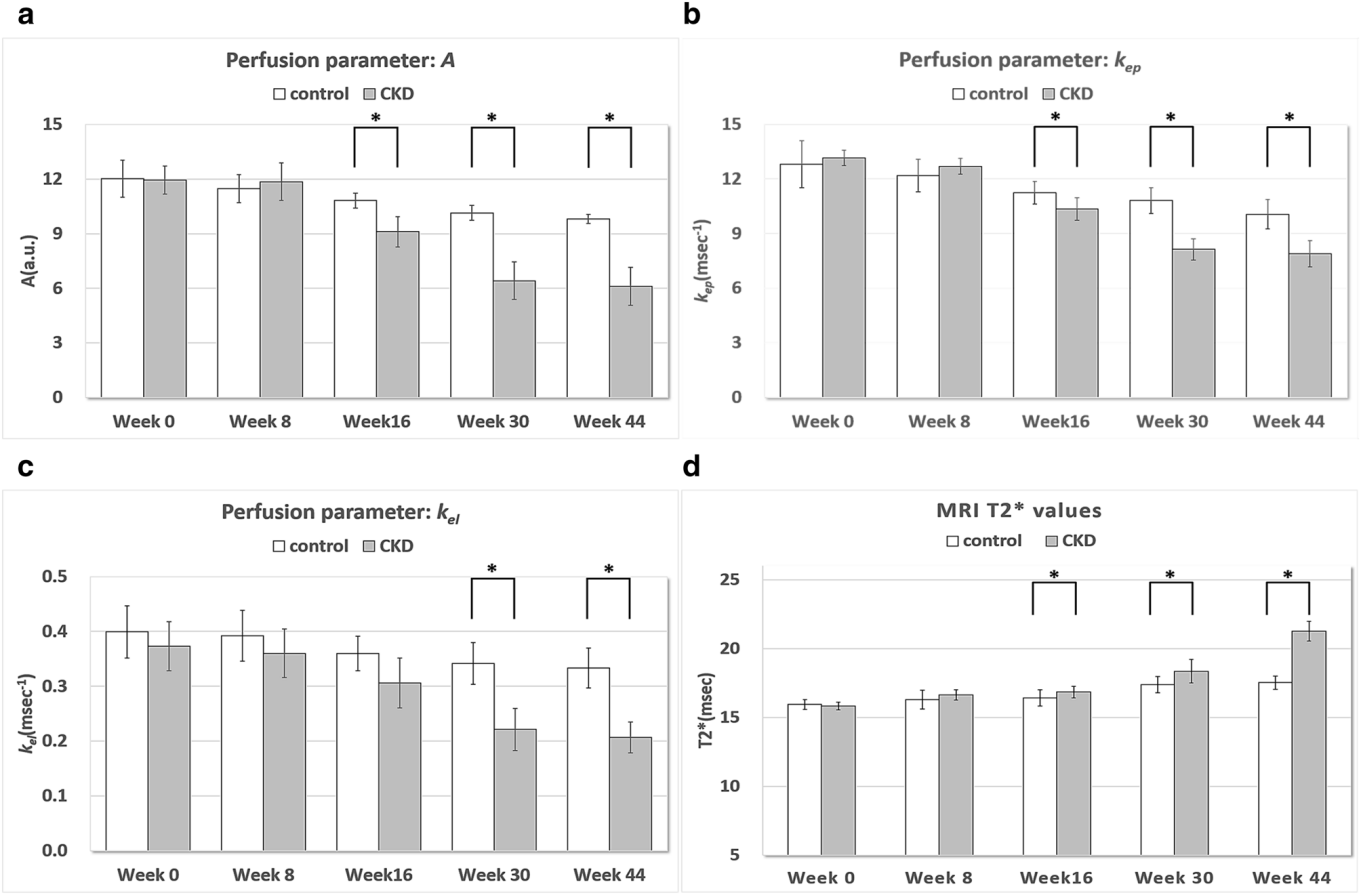
Bar charts showing the three perfusion parameters and MRI T2* values for IPFP of the control and CKD groups. The perfusion parameters (**a**) *A* (unit: a.u.), (**b**) *k*_*ep*_ (unit: min^−1^), and (**c**) *k*_*el*_ (unit: min^−1^) and the MRI T2* value (**d**) were measured in all rats at weeks 0, 8, 16, 30, and 44. Asterisks indicate significant differences (*p* < 0.05). Regarding (**a**) and (**b**) respectively perfusion parameters *A* (blood volume) and *k*_*ep*_ (permeability), significantly decreasing values can be found from week 16 to 44 in the CKD group. Regarding wash out parameter *k*_*el*_ (**c**), decreasing values were observed at 30 and 44 weeks. (**d**) MRI T2* values were significantly higher from week 16 to 44 in the CKD group (*p* < 0.05).

**Figure 2:**
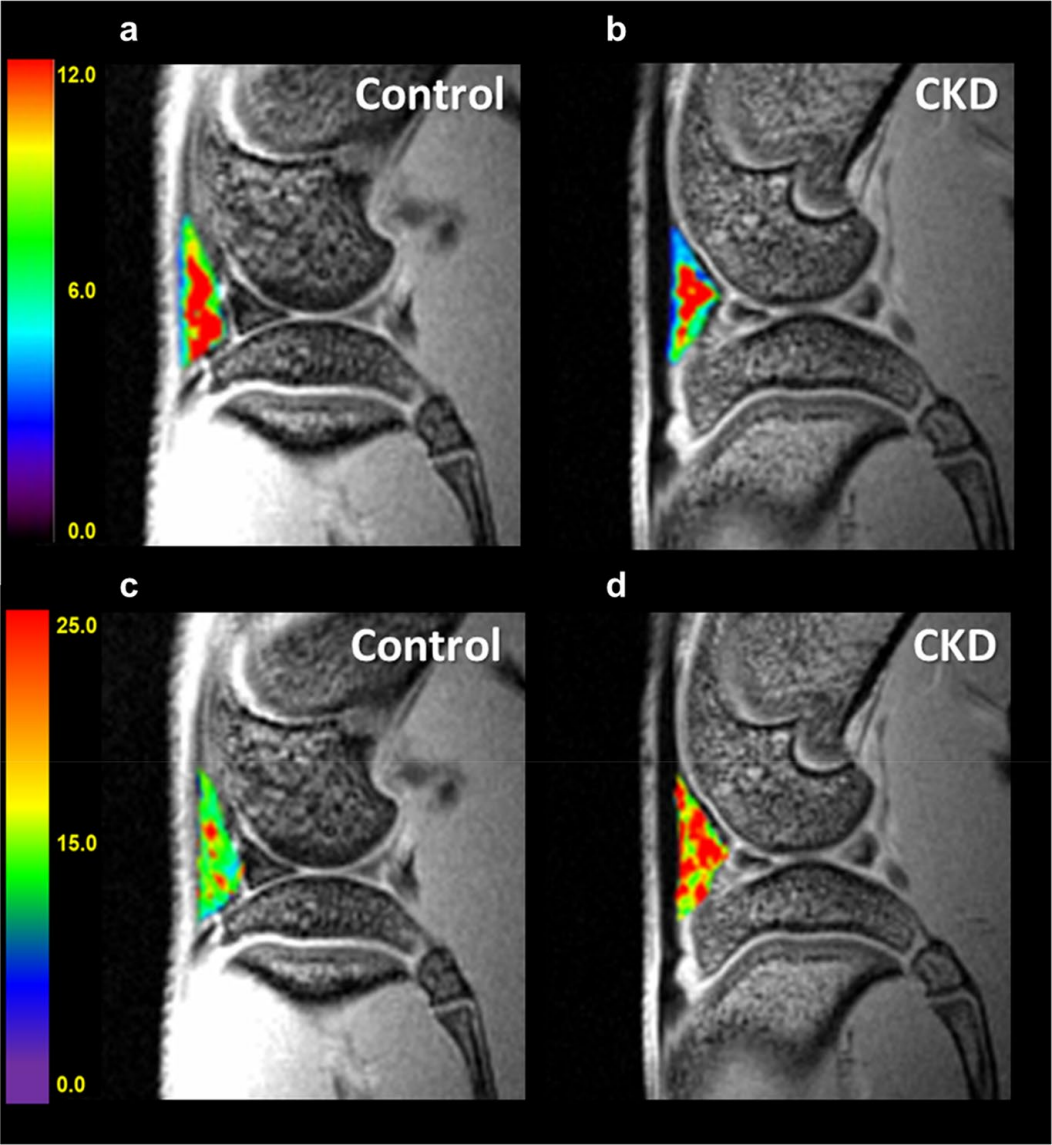
The demonstrated perfusion parameter *A* and MRI T2* maps of infrapatellar fat pad in control and CKD rats. The DCE-MRI (amplitude *A*) maps of infrapatellar fat pad in both groups. The color-coded images show significant hypo-perfusion in the CKD group (**b**) as compared to the control group (**a**), especially in the outer margin. Compared to the control group (**c**), the CKD group (**d**) had significantly higher T2* values (*p* < 0.05).

Multiple linear regression was evaluated using the GEE method. At week 0, no significant between-group difference was found in the three perfusion parameters *A, k*_*el*_, and *k*_*ep*_ (*p* values 0.387 to 0.888). In the control group, perfusion parameters (*A* and *k*_*el*_) at 8, 16, 30, and 44 weeks were statistically significantly decreased compared with their baseline values (*p* values 0.001 to < 0.001). Another perfusion parameter *k*_*ep*_ was significantly decreased at 16, 30, and 44 weeks (*p* values ranged from 0.002 to < 0.001). As compared to the control group, the CKD group demonstrated significantly lower perfusion parameters (*A* and *k*_*ep*_) at 16, 30, and 44 weeks (*p* values 0.028 to < 0.001; Fig. 1a, b, Tables 2 and 3). Representative images can be seen in Fig. 2a,b. A significant decline can be observed in *k*_*el*_ at 30 and 44 weeks (all *p* values < 0.001; Fig. 1c, Table 4).

**Table 2:** Comparisons of perfusion parameter *A* between control and CKD groups over 44 weeks by GEE multiple linear regression.

Parameters	B	SE	95% Wald CI		*p *value
**Parameter A**					
Intercept	12.017	0.4699	11.096	12.938	**< 0.001**
CKD versus Control	− 0.083	0.5912	− 1.242	1.075	0.888
**Compare the changes at each time point to week 0 in control group**					
[Week = 44] versus [Week = 0]	− 2.203	0.4733	− 3.131	− 1.276	**< 0.001**
[Week = 30] versus [Week = 0]	− 1.882	0.3321	− 2.533	− 1.231	**< 0.001**
[Week = 16] versus [Week = 0]	− 1.198	0.3637	− 1.911	− 0.485	**0.001**
[Week = 8] versus [Week = 0]	− 0.547	0.2088	− 0.956	− 0.137	**0.009**
**Compare the changes between CKD and control groups at each time point**					
[Week = 44]	− 3.615	0.8864	− 5.352	− 1.878	**< 0.001**
[Week = 30]	− 3.637	0.7939	− 5.193	− 2.081	**< 0.001**
[Week = 16]	− 1.630	0.5196	− 2.648	− 0.612	**0.002**
[Week = 8]	0.463	0.5022	− 0.521	1.448	0.356

B: regression coefficient.

**Table 3:** Comparisons of perfusion parameter *k*_*ep*_ between control and CKD groups over 44 weeks by GEE multiple linear regression.

Parameters	B	SE	95% Wald CI		*p* value
**Parameter k**_***ep***_					
Intercept	12.807	0.5961	11.638	13.975	**< 0.001**
CKD versus Control	0.357	0.6274	− 0.873	1.586	0.570
**Compare the changes at each time point to week 0 in control group**					
[Week = 44] versus [Week = 0]	− 2.747	0.5628	− 3.850	− 1.644	**< 0.001**
[Week = 30] versus [Week = 0]	− 1.998	0.4180	− 2.818	− 1.179	**< 0.001**
[Week = 16] versus [Week = 0]	− 1.565	0.5153	− 2.575	− 0.555	**0.002**
[Week = 8] versus [Week = 0]	− 0.627	0.3932	− 1.397	0.144	0.111
**Compare the changes between CKD and control groups at each time point**					
[Week = 44]	− 2.523	0.6794	− 3.855	− 1.192	**< 0.001**
[Week = 30]	− 3.030	0.5418	− 4.092	− 1.968	**< 0.001**
[Week = 16]	− 1.250	0.5704	− 2.368	− 0.132	**0.028**
[Week = 8]	0.153	0.4359	− 0.701	1.008	0.725

B: regression coefficient.

**Table 4:** Comparisons of perfusion parameter *k*_*el*_ between control and CKD groups over 44 weeks by GEE multiple linear regression.

Parameters	B	SE	95% Wald CI		*p *value
**Parameter k**_***el***_					
Intercept	0.399	0.0221	.356	0.443	**< 0.001**
CKD versus Control	− 0.026	0.0305	− 0.086	0.033	0.387
**Compare the changes at each time point to week 0 in control group**					
[Week = 44] versus [Week = 0]	− 0.066	0.0148	− 0.095	− 0.037	**< 0.001**
[Week = 30] versus [Week = 0]	− 0.058	0.0119	− 0.081	− 0.034	**< 0.001**
[Week = 16] versus [Week = 0]	− 0.040	0.0115	− 0.062	− 0.017	**0.001**
[Week = 8] versus [Week = 0]	− 0.007	0.0030	− 0.013	− 0.001	**0.016**
**Compare the changes between CKD and control groups at each time point**					
[Week = 44]	− 0.100	0.0256	− 0.151	− 0.050	**< 0.001**
[Week = 30]	− 0.094	0.0216	− 0.136	− 0.052	**< 0.001**
[Week = 16]	− 0.027	0.0235	− 0.073	0.019	0.249
[Week = 8]	− 0.006	0.0167	− 0.038	0.027	0.734

B: regression coefficient.

### MRI T2* analysis

As shown in Fig. 1d and Table 5, multiple linear regression using the GEE method showed no significant between-group difference at week 0 (*p* value = 0.592). In the control group, no significant difference at week 8 (*p* values = 0.16) but increased MRI T2* value at 16, 30, and 44 weeks were observed compared to baseline values (*p* value 0.007 to < 0.001). The MRI T2* values in the CKD group, compared to those in the control group, were not significantly different at week 8 (*p* values = 0.111) but significantly higher at 16, 30, and 44 weeks (*p* values 0.024 to < 0.001). Higher signal intensity is shown in the MRI T2* map presented in Fig. 2c,d.

**Table 5:** Comparisons of T2* values between control and CKD groups over 44 weeks by GEE multiple linear regression.

Parameters	B	SE	95% Wald CI		*p *value
T2* values					
Intercept	15.937	.1708	15.602	16.271	< 0.001
CKD versus Control	− 0.115	0.2147	− 0.536	0.306	0.592
**Compare the changes at each time point to week 0 in control group**					
[Week = 44] versus [Week = 0]	1.582	0.1889	1.211	1.952	**< 0.001**
[Week = 30] versus [Week = 0]	1.437	0.2369	0.972	1.901	**< 0.001**
[Week = 16] versus [Week = 0]	0.487	0.1809	0.132	0.841	**0.007**
[Week = 8] versus [Week = 0]	0.355	0.2526	− 0.140	0.850	0.160
**Compare the changes between CKD and control groups at each time point**					
[Week = 44]	3.860	0.3320	3.209	4.511	**< 0.001**
[Week = 30]	1.093	0.4000	0.309	1.877	**0.006**
[Week = 16]	0.535	0.2377	0.069	1.001	**0.024**
[Week = 8]	0.455	0.2851	− 0.104	1.014	0.111

B: regression coefficient.

### Correlation analysis

**Figure 3:**
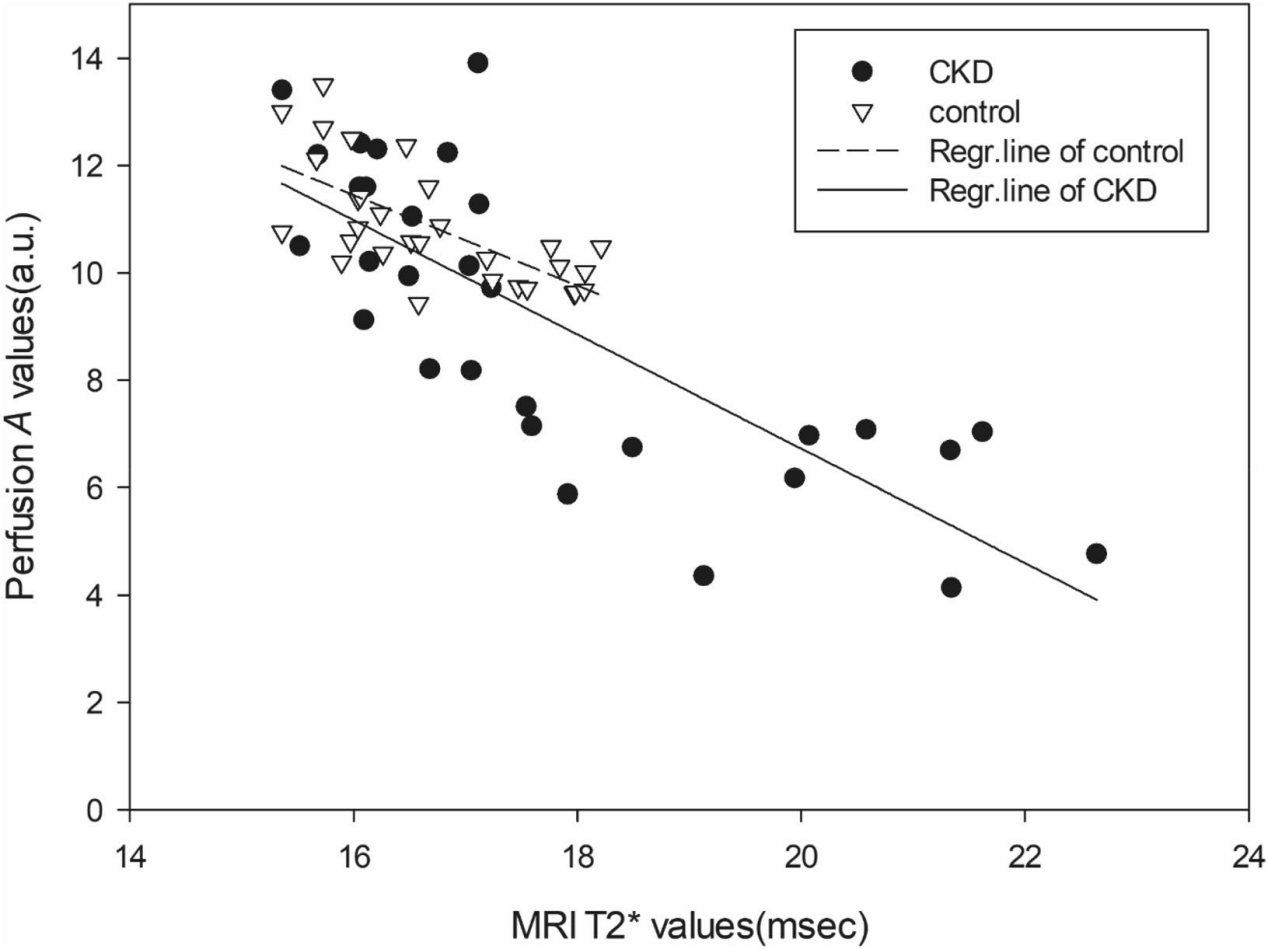
The correlation of perfusion parameter *A* (a.u.) and MRI-T2* value (msec) in control rats (inverted triangles) and CKD rats (black circles). All symbols represent measurements made in the right knee joint. Correlations between perfusion parameter *A* values and MRI-T2* values in the IPFP were significantly negative in the control and CKD groups (*p* < 0.001). The CKD group, compared to control group, had a higher negative correlation coefficient (ρ =  − 0.815) between perfusion parameter *A* and MRI-T2* (*p* < 0.001).

The DCE perfusion parameter *A* and MRI T2* values of infrapatellar fat pad were correlated for the control and CKD groups. The Spearman correlation coefficient between the perfusion parameter *A* and the MRI T2* value was ρ =  − 0.724 (*p* < 0.001) in the control group, indicating significant negative correlation, and ρ =  − 0.815 in the CKD group (*p* < 0.001; Fig. 3), indicating negative correlation of even greater significance.

### Histologic analysis

**Figure 4:**
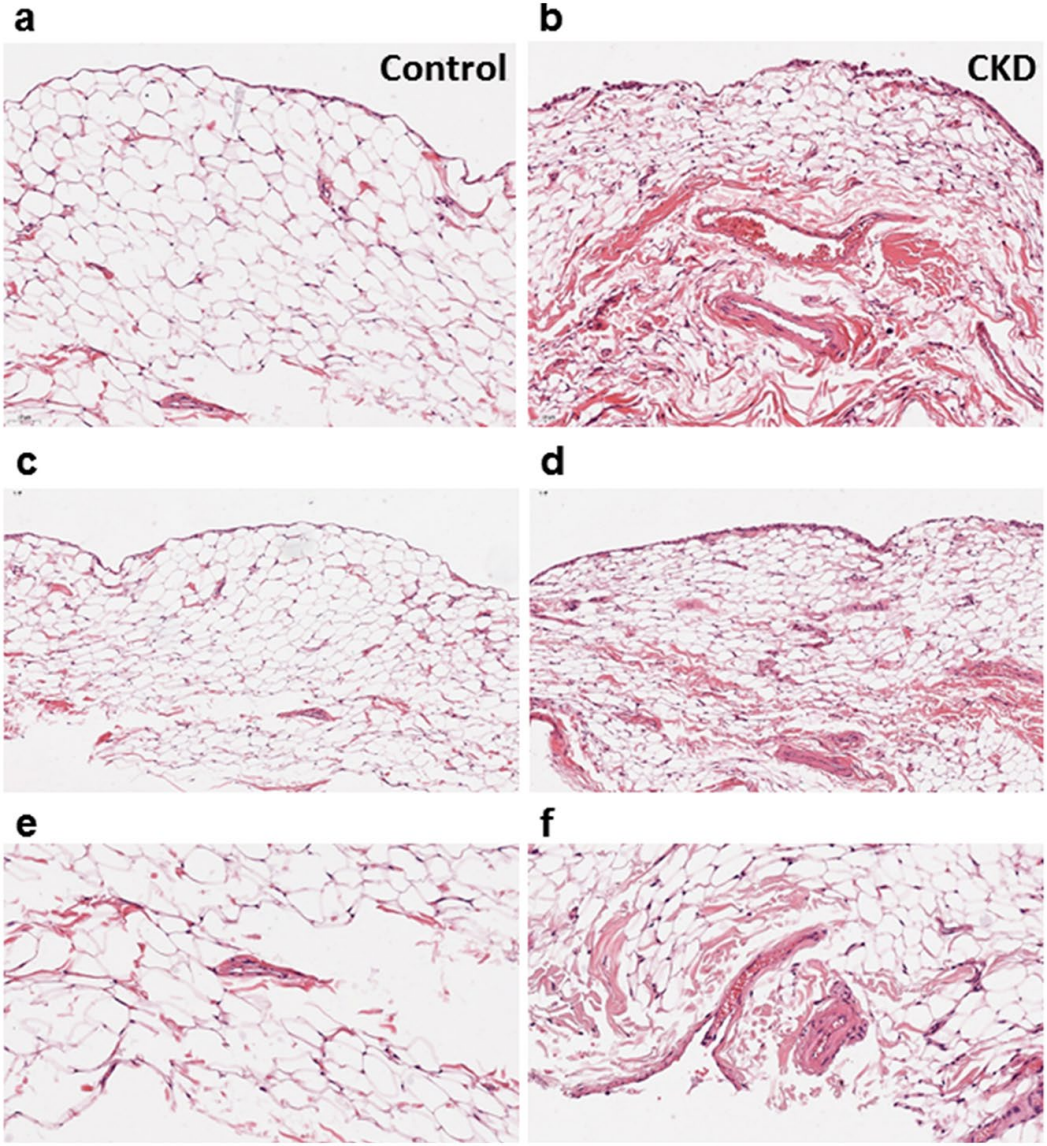
Histopathological staining of the infrapatellar fat pad in the control and CKD groups. A relatively increased vessel wall thickness of infrapatellar fat pad can be observed in the CKD group (**b**) as compared to control group (**a**). While fat pad tissues in the control group (**c**, **e**) have a relatively normal appearance, in the CKD group (**d**, **f**), they exhibit adipocyte degeneration with myxoid change. [(**a**–**d**) × 200, (**e**,**f**) × 400].

In hematoxylin and eosin stained sections, the artery of the infrapatellar fat pad demonstrated a normal appearance in the control group, but mildly increased wall thickness, intimal edema, and vascular smooth muscle hyperplasia in the CKD group (Fig. 4a,b). The fat pad adipose tissue had a normal appearance in the control group but was characterized by prominent synovial lining, increased fibrous septum material, small vessels proliferation, and irregular enlargement of adipocytes in the CKD group, reflecting myxoid degeneration of the infrapatellar fat pad (Fig. 4c–f).

## Discussion

A relationship between hypoperfusion of the IPFP and its degeneration was identified in this animal CKD study. Change in perfusion parameters and T2* values during CKD progression was demonstrated. The DCE-MRI and MRI T2* changes were confirmed histopathologically. Furthermore, we demonstrated the vasculopathy of the IPFP and the feasibility of using longitudinal DCE-MRI and MRI T2* values in a rodent 5/6 nephrectomy model. For human subjects, this information could be potentially useful for in vivo investigation of knee osteodystrophy in CKD-MBD.

The IPFP is thought to play a role in the initiation and progression of knee osteoarthritis^23^. Any form of impingement such as acute or repetitively chronic microtrauma and synovitis will cause fat pad enlargement and inflammation. Except for OA-related biomechanical impingement, known as Hoffa’s disease, flow deficit is considered the possible contributor to injury^24^. In DCE-MRI with pharmacokinetic model analysis, the perfusion parameter *A* is a composite factor that reflects intrinsic vascularization, flow resistance, and interstitial volume^17,25^. The decrease in parameter *A* value in our results may reflect impaired vascularization caused by atherosclerotic change in the supplying vessels. The *k*_*el*_ is a constant that mirrors the washout rate of contrast medium from the interstitium. In our results, the decreasing elimination constant *k*_*el*_ may indicate impaired renal function due to venous obstruction in the 5/6 nephrectomy model. The permeability parameter *k*_*ep*_ is the rate constant of contrast medium transfer between the interstitial and the plasma compartments. In our study, the significant decrease in *k*_*ep*_ from week 16 may imply vascular dysfunction in CKD^26^. Although no previous study ever described the pathophysiology of vascular insufficiency in the fat pad in CKD, fat pad vascularity may play a role in the progression of CKD.

Ballegaard et al. demonstrated the feasibility of DCE-MRI to quantify IPFP inflammation in arthritic knees^19^. Besides, MRI T2* techniques utilize intrinsic water as a surrogate marker to investigate the integrity of the extracellular matrix^27,28^. In the present study, MRI T2* was a good imaging biomarker to monitor sequential change in the IPFP of our 5/6 nephrectomy model as well. An increase in MRI T2* relaxation time from week 16 may reflect extracellular matrix changes resulting from small vessel proliferation with mild myxoid change^29^. Although the value of MRI T2* in the IPFP is rarely reported in prior study, increased T2* values within the fat pad indicate inflammation, edema, or hemorrhage in Hoffa’s disease^14,30^. The impact of flow deficit is not as direct in CKD-related OA as in Hoffa’s fat pad impingement, however, the responses of the two diseases (when assessed by MRI T2*) are identical. Reduced perfusion of IPFP may possibly lead to subtle ischemia, and induce the abnormal distribution of substance P, adipose tissue inflammation, and edema eventually^31,32^. Moreover, MRI T2* increased simultaneously when DCE parameter *A* decreased at week 16, which demonstrates the relationship of changed IPFP blood flow to fibrosis and degeneration of adipocytes. Histopathological examination confirmed the presence of fat pad degeneration.

Additionally, in the progression of CKD, age effects should be considered especially in vascular disease. In the present report, three perfusion parameters (*A*, *k*_*el*_, and *k*_*ep*_) were significantly decreased at week 16, 30, and 44 in control rats, which indicates that perfusion declines in aging animals. Hence, the relationship we observed between hypoperfusion of infrapatellar fat pad and structural change in CKD rats was age-independent. In week 16 (in rats aged 24 weeks), the deficit in blood flow may be caused by restriction of feeding. To allow all rats of different ages to fit into the MR bore, which is limited in size, we restricted food intake to prevent the rats from becoming overweight. A possible change in vascularity due to smaller IPFP size may exist^33,34^. However, the control and CKD groups were fed in the same manner, and the feeding conditions should be the same. In summary, non-mechanical flow changes in CKD caused degenerative changes in the IPFP. The possible mechanism may be direct chronic hypoxia of adipocytes resulting from flow deficiency. The highly vascularized IPFP is an endocrine tissue that secretes adipokines^35^, therefore it is susceptible to hemodynamic change^36^. Poor oxygenation and nutrition might further disturb adipokine secretion, disrupt distribution of substance P, and cause fat pad inflammation and degeneration. In the future, further molecular studies should address the pathogenesis of vascular insufficiency in the fat pad.

Several limitations exist in our results. Even though atherosclerosis is associated with many risk factors such as aging, hypertension, diabetes, hyperphosphatemia, hyperparathyroidism, and hypervitaminosis D, our prospective animal study did not control for many factors such as parathyroid hormone and other factors. Second, the study was performed in male rats, and gender differences should be considered. Third, in order to reduce the number of animals sacrificed, a sham group was not included in our experiment. Fourth, the IPFP inflammation is a source of anterior pain in osteoarthritis. Its linkage to anterior pain has not been investigated in our animal model. Fifth, the size and various parts of the IPFP were not evaluated in our study. The vertical and horizontal clefts were not excluded in ROI analysis, which will introduce bias into the results. Sixth, quantitative immunohistochemical evaluation of specific biomarkers was not done.

In conclusion, hypoperfusion of the infrapatellar fat pad was associated with CKD, as evidenced by changes in DCE values, MRI T2^*^ values, and histopathologic features in a 5/6 nephrectomy model. The age-independent decrease in three DCE-MRI parameters (values *A*, *k*_*ep*_ and *k*_*el*_) and increase in MRI T2^*^ values were progressive over time in CKD. DCE MRI and MRI T2* could be reliable imaging biomarkers for monitoring the progression of CKD-related infrapatellar fat pad hypoperfusion.

## Methods

### Ethical statement for this experimental study

Study experiments were performed in accordance with the guidance for the Care and Use of Laboratory Animals of the U.S. National Institutes of Health. Our study was carried out in compliance with the ARRIVE guidelines. The study protocol was reviewed and approved by the Committee on the Ethics of Animal Experiments of the National Defense Medical Center (Permit Number: IACUC-12-259), Taipei, Taiwan. Isoflurane anesthesia was used to perform all animal procedures and 3R principles were followed in all animal experiments.

### Animal experiments

**Figure 5:**
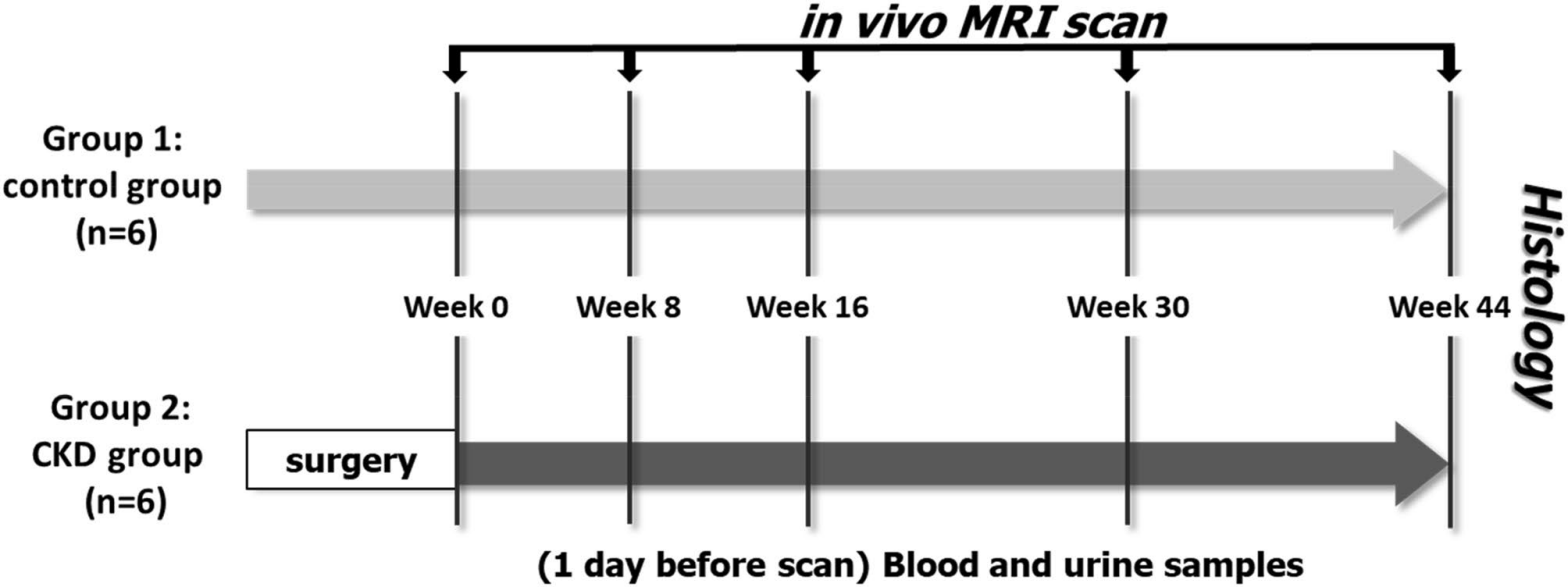
Flow chart of the experimental design. In vivo MRI scan (including DCE-MRI and MRI-T2*) was performed in Groups 1 and 2 at week 0, 8, 16, 30, and 44 after surgery in Group 2, indicated by arrows. At week 44, all rats were sacrificed and underwent histological analyses.

Twelve male Sprague–Dawley rats at 8 weeks of age, weighing about 300 g, were arbitrarily allocated into two groups (*n* = 6 for each group): rats without any surgery or treatment procedure served as group 1 (the control group) and rats with CKD induced by two-step 5/6 nephrectomy served as group 2 (the experimental group)^37^. At different times after kidney surgery, urine and blood samples and right knee MR images were acquired. The protocol is illustrated in Fig. 5. The right knees of all rats were longitudinally scanned by MRI, and serial changes in the infrapatellar fat pad were assessed at 0, 8, 16, 30, and 44 weeks by DCE-MRI and MRI T2* mapping.

All rats were anesthetized by inhalation of a 5% isoflurane mix administered at an oxygen flow rate of 5 L/min and placed into a body holder. A maximum of 3 rats were housed in one cage in a specific pathogen-free environment. The animals were maintained under a 12 light/12 dark cycle, 21 ± 2 °C temperature, and 50 ± 5% humidity and fed 5053-PicoLab Rodent Diet 20 with restriction. All animals were carefully monitored prior to, during, and after the experiment. The MRI experiment was performed in a 4.7 T magnet (Bruker, Ettlingen, Germany) equipped with a receiver quadrature surface coil, which was placed above the right knee joint, while 1–2% isoflurane at 2 L/min oxygen flow was provided to maintain anesthetic depth.

### DCE-MRI of infrapatellar fat pad

DCE-MRI was done by utilizing a fast gradient recalled echo (GRE) sequence with TR/TE = 115.5/4.47 ms, NEX = 1, matrix size = 128 × 128, FOV = 30 × 30 mm^2^, slice thickness = 0.65 mm, flip angle = 60°, bandwidth = 37.9 kHz, and acquisition time = 19 min 43 s. After 0.2 mmol/kg gadobutrol bolus injection manually (Gadovist; Bayer, Berlin, Germany), 7 sagittal slices and 80 serial dynamic images were acquired.

**Figure 6:**
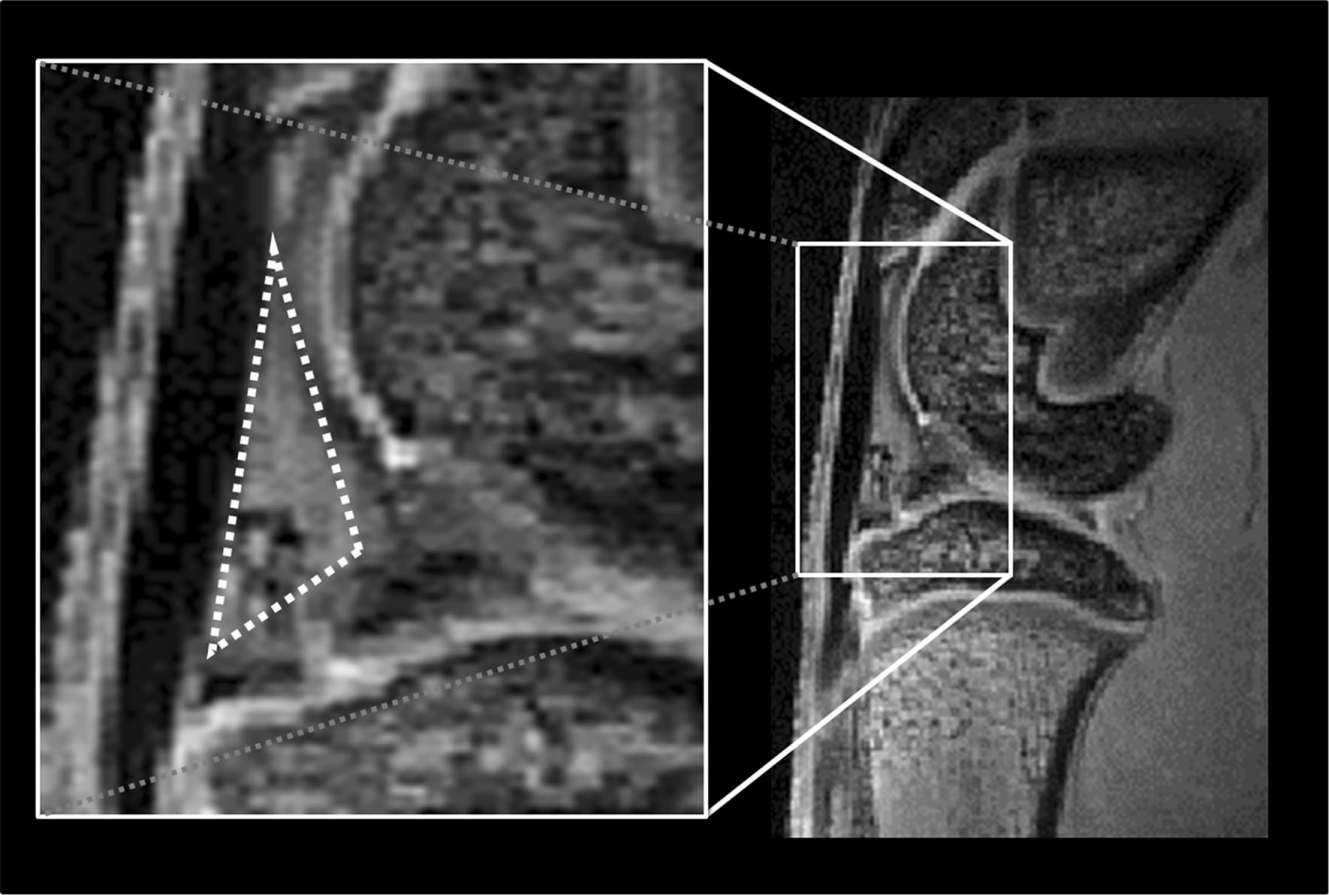
The selection of a ROI in the infrapatellar fat pad. The infrapatellar fat pad ROIs in DCE-MRI and MRI T2* were positioned using the sagittal image with the largest fat pad available. A triangular ROI under the infrapatellar tendon and near the anterior femur and tibial plateau was drawn manually without including the anterior horn of the meniscus of the knee joint.

The imaging data were post-processed by MIStar software (Apollo Technology, North Melbourne, Australia). Three quantitative perfusion parameters, amplitude (*A*), elimination constant (*k*_*el*_), and permeability rate constant (*k*_*ep*_) were estimated from a pixel-wise analysis of the infrapatellar fat pad ROI as shown in Fig. 6. The signal intensities within the ROI were averaged and transformed to concentration–time data^25^. Afterward, the concentration–time data (Ct) were fitted to the Brix pharmacokinetic model by using a nonlinear least-square curve fitting algorithm^17^. Three perfusion parameters were generated based on the model and obtained from the fitted time-signal intensity curve.

### MRI T2* of infrapatellar fat pad

MRI T2* was measured by a fast GRE sequence with shorter minimal echo time (TE). Seven sagittal slices were collected using a multi-slice and multi-echo fast GRE sequence with TR = 600 ms, 8 TEs = 3.5, 8.5, 13.5, 18.5, 23.5, 28.5, 33.5, and 38.5 ms, NEX = 12, matrix size = 256 × 192, FOV = 30 × 30 mm^2^, slice thickness = 0.65, flip angle = 30°, bandwidth = 69.4 kHz, and acquisition time = 30 min 43 s.

The single-exponential, least-squares curve-fitting method was chosen to analyse the MRI T2* relaxation time of the infrapatellar fat pad and reduce fitting errors in low signal-to-noise images^38^. The spin density (M_0_) and apparent transverse relaxation time (T2*) were determined after fitting the signal magnitude to a mono-exponential decay model. The infrapatellar fat pads were manually drawn from first-frame MRI T2* images. To reduce the discrepancies in the manual positioning of the ROIs, the ROIs were drawn by two image analysts (CYW: 21 years of experience, SWC: 11 years of experience) and were confirmed by a musculoskeletal radiologist with over 30 years of experience (GSH). Results shown in this study are the mean of two measurements.

### Histologic analysis

At week 44, all rats were sacrificed for removal of the right knee joint. The samples were fixed in 10% buffered formalin for 12 h, and decalcified at room temperature in a rapid decalcifier with ethylene-diamine-tetra-acetic acid (EDTA) for 24 h (Nihon Shiyaku Industries, Osaka, Japan). The IPFP of the knee joint was removed, paraffin-embedded, and cut into 3-µm slices for hematoxylin and eosin (HE) staining. The vessels and IPFP tissues of both groups were evaluated to verify the findings of DCE-MRI and MRI T2*. All histologic analyses were carried out by a senior pathologist YJP (with 21 years of experience) and confirmed by HSL (with over 30 years of experience).

### Statistical analysis

The differences in urine and blood sample values between the control and CKD groups were analysed by using the Student t-test. The mean value and 95% confident interval (CI) of DCE-MRI and MRI T2* values were calculated in both groups. To take into account the within-subjects’ dependency (due to repeated measurement), the GEE (generalized estimating equation) method’s multiple linear regression model was used, with groups (control and CKD), time (0, 8, 16, 30, and 44 weeks), and their interaction terms included in the model to compare between-group differences in the changes in longitudinal DCE-MRI and MRI T2* from week 0^39^. The correlation between MR T2* values and perfusion parameter *A* was further analysed by using the Spearman correlation coefficients. The reproducibility of the DCE and MRI T2* measurements was assessed by the inter-class correlation coefficient (ICC), and the values > 0.75 were interpreted as good. All analyses were done by using the SPSS v26.0 software (SPSS Inc., Chicago, IL, USA). A *p* value of < 0.05 was regarded as statistically significant.
